# Complement factor H in host defense and immune evasion

**DOI:** 10.1007/s00018-016-2418-4

**Published:** 2016-12-10

**Authors:** Raffaella Parente, Simon J. Clark, Antonio Inforzato, Anthony J. Day

**Affiliations:** 1 Humanitas Clinical and Research Center, Via Manzoni 56, Rozzano, 20089 Milan, Italy; 2 Division of Evolution and Genomic Medicine, School of Biological Sciences, Faculty of Biology, Medicine and Health, University of Manchester, Oxford Road, Manchester, M13 9PT UK; 3 Department of Medical Biotechnologies and Translational Medicine, University of Milan, Via Vanvitelli 32, 20129 Milan, Italy; 4 Wellcome Trust Centre for Cell-Matrix Research, Division of Cell-Matrix Biology and Regenerative Medicine, School of Biological Sciences, Faculty of Biology, Medicine and Health, University of Manchester, Oxford Road, Manchester, M13 9PT UK

**Keywords:** Complement cascade, Complement factor H, Glycan markers, Inflammatory diseases, Cancer immunology, Innate immunity

## Abstract

Complement is the major humoral component of the innate immune system. It recognizes *pathogen*- and *damage*-*associated molecular patterns*, and initiates the immune response in coordination with innate and adaptive immunity. When activated, the complement system unleashes powerful cytotoxic and inflammatory mechanisms, and thus its tight control is crucial to prevent damage to host tissues and allow restoration of immune homeostasis. Factor H is the major soluble inhibitor of complement, where its binding to *self markers* (i.e., particular glycan structures) prevents complement activation and amplification on host surfaces. Not surprisingly, mutations and polymorphisms that affect recognition of *self* by factor H are associated with diseases of complement dysregulation, such as age-related macular degeneration and atypical haemolytic uremic syndrome. In addition, pathogens (i.e., *non*-*self*) and cancer cells (i.e., *altered*-*self*) can hijack factor H to evade the immune response. Here we review recent (and not so recent) literature on the structure and function of factor H, including the emerging roles of this protein in the pathophysiology of infectious diseases and cancer.

## Introduction

**Fig. 1:**
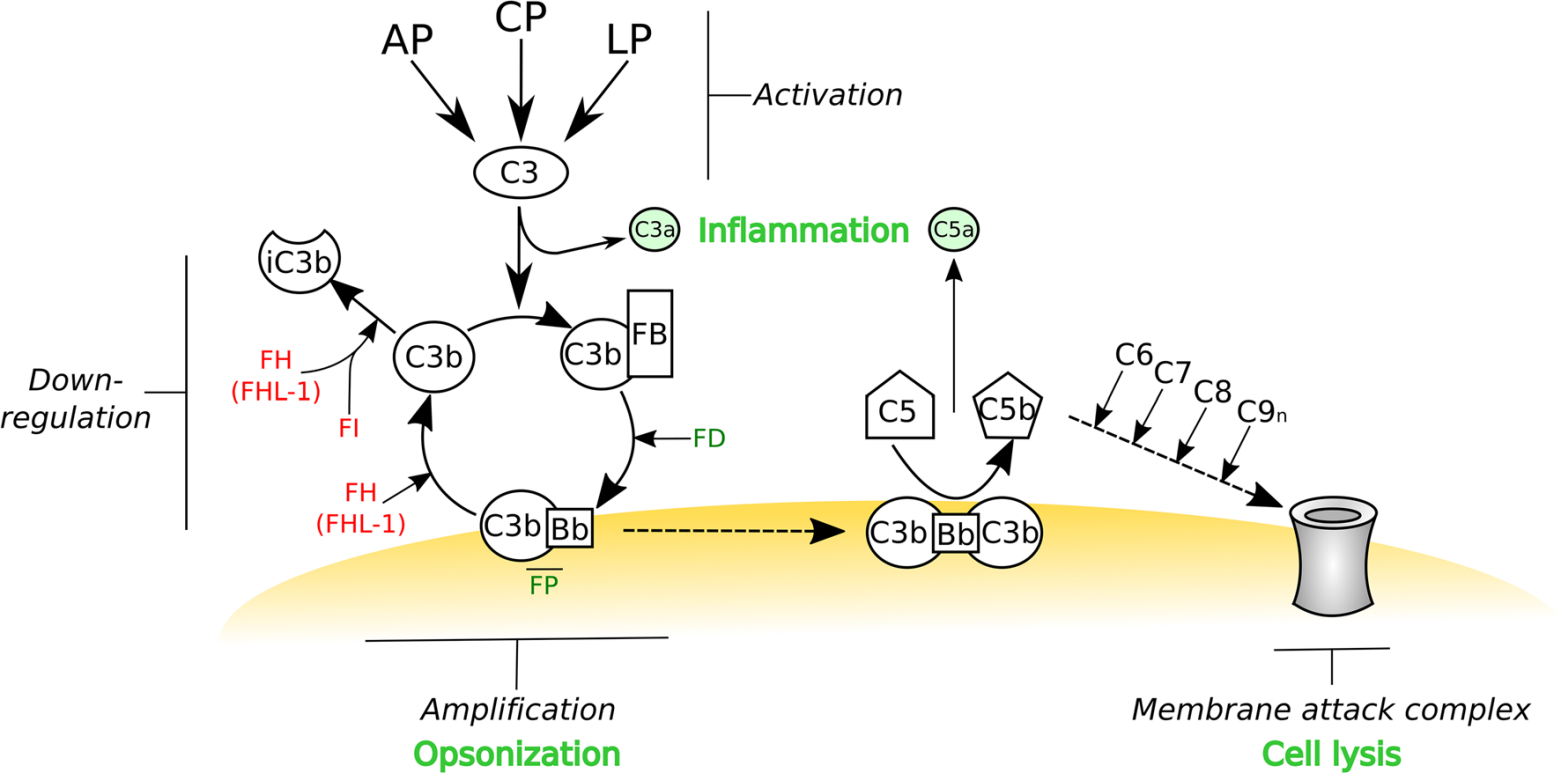
The activation, amplification and regulation of the complement system. The complement system can be activated by three different pathways: the alternative pathway (AP), classical pathway (CP) and lectin pathway (LP). While the AP is constitutively active and undergoes a constant *tick over*, the CP and LP are triggered by antibody- and carbohydrate-mediated recognition mechanisms, respectively. Regardless of how this protein cascade is initiated, it is the AP that amplifies the complement system; e.g. if the AP C3 convertase is formed on an activator surface. All three pathways lead to the conversion of C3 into C3b, which can become covalently attached (via a thioester) to any nearby nucleophile (e.g., a hydroxyl or amine group on a surface); C3 cleavage releases the small protein fragment C3a, a potent anaphylatoxin. The remaining C3b can associate with factor B (FB), which is itself cleaved by factor D (FD). This forms the AP C3 convertase (i.e., C3bBb) where this is stabilized by factor P (FP). If this convertase is not deactivated then any free C3 in the vicinity will be converted into C3b and thus begins a positive feedback cycle, referred to as the amplification loop. If left to run unchecked, this will lead to the opsonization of the target surface (with C3b) and the formation of the AP C5 convertase (i.e., C3bBbC3b), which represents the initiation of the terminal pathway of complement and the subsequent formation of the membrane-attack complex (MAC) that can lead to lysis (e.g., of bacteria). Host cells have a number of cell surface proteins capable of down-regulating the complement cascade that can either dissociate the AP C3 convertase, known as decay acceleration activity, or act as cofactors for the proteolytic cleavage, by factor I (FI), to inactive C3b (iC3b). The soluble C3b-binding protein factor H (FH) can associate with host cells via the recognition of glycan markers of *self* and thereby down-regulate complement through its decay accelerating and cofactor activities. FH (and the truncated FHL-1 product of the *CFH* gene) can also recognize *self *markers on acellular structures such as the extracellular matrix, and in this context FH/FHL-1 are the only negative regulators of the complement AP pathway. Therefore, FH and FHL-1 have a pivotal role in the prevention of complement activation in host tissues. This schematic is modified from [184]

Innate immunity comprises a humoral (macromolecular) and a cellular arm [1, 2]. The complement system is a major component of the former, comprised of a cascade of more than 30 proteins, which is activated via three distinct pathways: alternative (AP), classical (CP) and lectin (LP) [3]; see [4] for complement nomenclature. Activation of the classical and lectin pathways is restricted to the recognition of immune complexes and *non*-*self* carbohydrates, respectively, by soluble pattern recognition molecules/receptors (PRMs/PRRs), including the C1q moiety in the CP, and mannose-binding lectin (MBL) and ficolins (both in LP) [3, 5]. Associated serine proteases (i.e., C1r/C1s in CP and mannan-binding lectin-associated serine proteases (MASPs) in LP) then cleave C4 and C2, thus generating C4b and C2a fragments that non-covalently associate to form the CP/LP C3 convertase (C4bC2a). This enzyme complex catalyzes the hydrolysis of C3 to C3a and C3b, where the latter contains a highly reactive thioester that can form a covalent bond with a nucleophile, e.g. on a nearby cell or in the extracellular matrix. Surface-bound C3b can then form a complex with factor B (FB), which is cleaved by factor D to Bb, leading to the formation of the alternative pathway C3 convertase (C3bBb), which is stabilized by factor P (FP; also termed properdin) and catalyzes further hydrolysis of C3. Therefore, as shown in Fig. 1, when the AP C3 convertase forms on an *activating* surface, lacking an appropriate level of complement inhibitors, the AP can act as an amplification loop for the complement system, regardless of how this is initially triggered (see below and [6]). AP activation also occurs in the absence of a recognition event through a *tick over* mechanism. Here, C3 undergoes non-enzymatic hydrolysis of the thioester bond to generate C3(H_2_0) [7] that can bind FB, which is itself cleaved by FD to make a fluid-phase C3-convertase (i.e., C3(H_2_0)Bb). This convertase is capable of cleaving further C3 molecules to C3b that can then bind covalently to available surfaces (via an ester bond as described above) and lead to amplification of complement if left unchecked.

Thus all three pathways converge in the formation of C3 convertases and in the deposition of C3b that opsonizes pathogens (and host cell debris) and promotes their phagocytosis [6]. Moreover, by binding C3b, the C3 convertases can be converted to C5 convertases (i.e. C4bC2aC3b and C3bBbC3b for the CP/LP and AP, respectively) leading to the formation of the membrane-attack complex (MAC, C5b–C9n) that can cause lysis of the target cells (see Fig. 1); the anaphylatoxins C3a and C5a, generated as by-products of convertase formation (i.e. C3 and C5 cleavage), act as potent chemoattractants and activators of immune cells. In addition to these well established effector functions of complement (i.e., being opsonic, lytic and pro-inflammatory), it has become increasingly apparent that this protein cascade also plays key roles in selection, maintenance and activation of B lymphocytes, where C3 and its proteolytic fragments are necessary to elicit a robust antibody response in antigen-primed B cells [8]. Furthermore, an involvement of complement is emerging in T cell immunity, with regard to activation, polarization and survival of T cell subsets [6, 9, 10]. Therefore, the biological activities of complement extend beyond recognition/disposal of invading pathogens and encompass mechanisms in both innate and adaptive immunity, where the complement system has been described as a *natural adjuvant* and a modulator of the adaptive response [11].

In the light of the pleiotropic functions of the complement system and, most importantly, the destructive potential of its activation (because of the potent amplification ability of the AP), complement requires a number of regulatory mechanisms that allow its tight control to confine it to appropriate pathogenic surfaces, and thereby prevent collateral damage to healthy host tissues [3]. Distinct phases of complement activation are indeed checked by fluid-phase and cell-associated inhibitors so that the final outcome represents an intricate balance between detection and destruction of *non*-*self* and minimization of damage to *self* [3, 6, 12]. In this regard, both C3b and C4b undergo proteolytic processing by factor I (FI), where this enzyme requires, as cofactors, either fluid-phase regulators, i.e. factor H (FH) and C4-binding protein (C4BP) for AP and CP/LP, respectively, or membrane proteins on the cell surface, i.e. complement receptor type 1 (CR1; CD35) and membrane cofactor protein (MCP; CD46). Also, the rate of formation and dissociation (decay) of the C3 (and C5) convertases is controlled by FH, decay-accelerating factor (DAF; CD55), CR1 and C4BP that all serve to reduce the amount of active convertase present; on the other hand, FP stabilizes the C3 and C5 convertases of the AP. In addition, the formation of the membrane-attack complex is inhibited by CD59 (also known as MAC-inhibitory protein) and vitronectin (S protein).

FH is the major regulator of the AP in solution phase, but also has a critical role in the inhibition of complement amplification on cells and in the extracellular matrix of host tissues due to its ability to recognize (and associate with) *self surfaces* (i.e., via glycan markers) to ensure that these are non-activating (see [13, 14]). In this regard, FH is amongst the most abundant complement components in human blood (with plasma concentrations ranging from 200 to 800 μg/ml; see [15]), where the liver is the major source of FH protein in vivo. However, FH is secreted by additional cell types including monocytes [16], fibroblasts [17], endothelial cells [18], platelets [19], and retinal pigment epithelial cells (RPE) [20, 21], that likely contribute to local titers of the protein in tissues [22]. Furthermore, the complement factor H (*CFH*) gene undergoes alternative splicing, where a truncated form of FH, termed factor H-like protein 1 (FHL-1) [23], can also be expressed locally, e.g. by RPE cells, as well as by the liver [21, 23]. Thus, systemic and locally secreted FH, most likely, both contribute to the regulation of complement activation in the context of immune surveillance [21, 24, 25].

Given the central role of FH in regulation of complement amplification (Fig. 1), proper functioning of this soluble inhibitor is crucial, as exemplified by human pathologies associated with FH, including those with mutations and polymorphisms in the *CFH* gene; e.g. typical and atypical hemolytic uremic syndrome (HUS and aHUS, respectively) [13, 26–28], age-related macular degeneration (AMD) [29–31] and dense deposit disease (DDD) [32, 33]. In this review, we discuss the structure and function of FH as a fundamental complement inhibitor and focus on its roles in complement-mediated diseases and the immune evasion strategies adopted by pathogens and cancer cells.

## Structural insights into the complement inhibitory activities of factor H

**Fig. 2:**
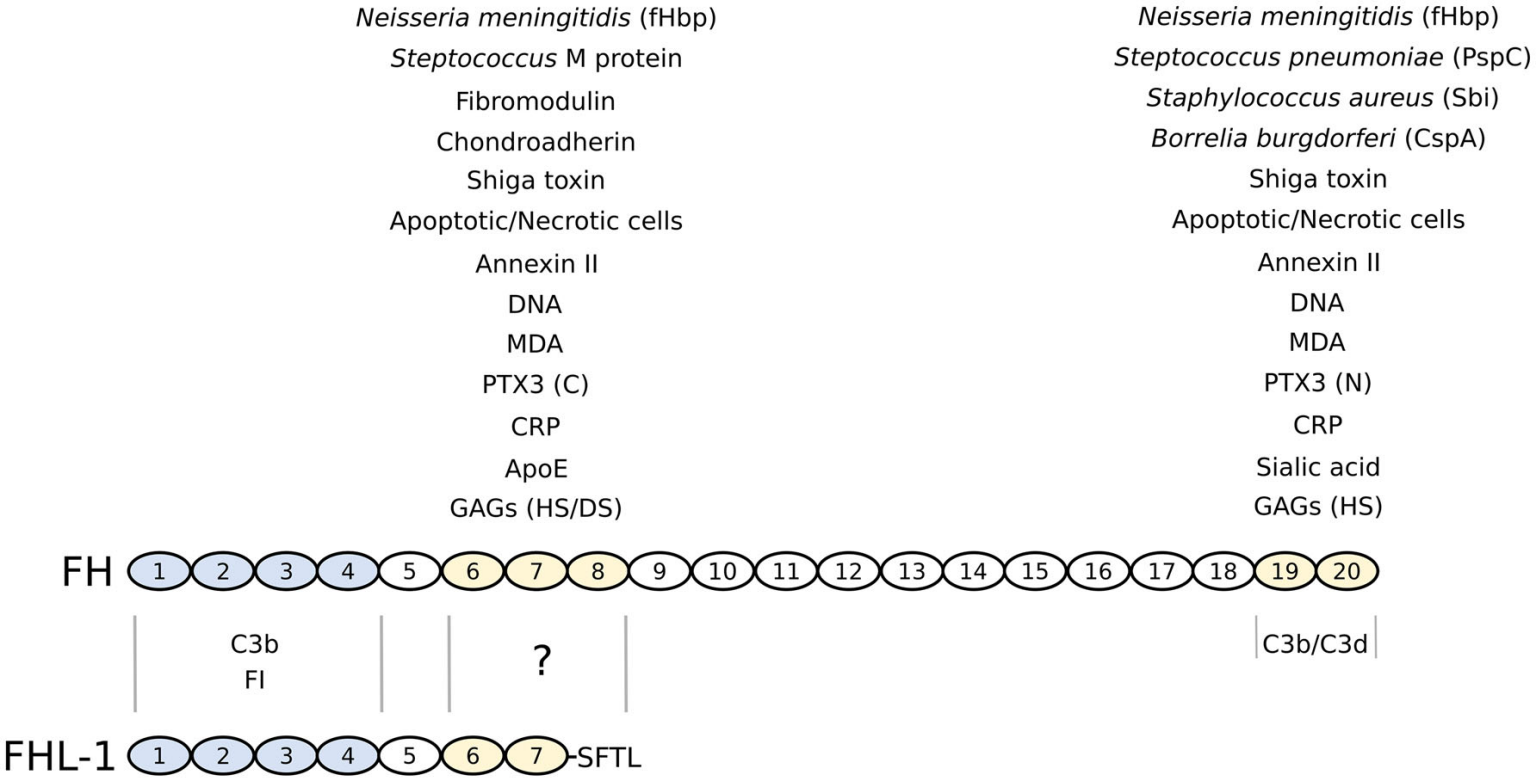
Domain structure and ligand-binding activities of factor H and FHL-1. Complement FH is comprised of twenty CCP modules, whereas FHL-1 shares the first seven of these followed by a unique four amino acid sequence (SFTL). The four N-terminal CCPs of FH/FHL-1 (colored *blue*) confer regulatory activity through mediating C3b and FI binding; C3b (via its C3d region) also binds to the C-terminal CCPs 19–20 of FH. The CCPs 6–8 and/or 19–20 regions (colored *yellow*) support the interactions with a wide variety of ligands including glycans (GAGs and sialic acid), pentraxins (CRP and PTX3; where the latter binds through the its C- and N-terminal domains, respectively), the lipid peroxidation product MDA, apoptotic/necrotic cells (e.g., via annexin II and DNA), extracellular matrix proteins (e.g., chondroadherin and fibromodulin), ApoE, shiga toxin from enterohaemorrhagic *E. coli*, and FH-binding proteins from various other bacteria. Although it is presumed that many of the ligands that bind FH via CCPs 6–8 will interact also with FHL-1, most of these have yet to be tested

The human FH gene (termed *CFH*) is located on chromosome 1q32 in the regulators of complement activation (RCA) gene cluster. This encodes an ~155 kDa glycoprotein comprised of 20 contiguous complement control protein (CCP) modules [23, 34] (Fig. 2), also known as short consensus repeats or sushi domains, which occur in many other complement and non-complement proteins [23, 35]; the alternatively spliced transcript, FHL-1 is composed of CCPs 1–7 followed by a unique 4-amino acid sequence. Each CCP consists of ~60 amino acids arranged in a typical β-sandwich fold (containing up to 8 β strands), which approximate to prolate ellipsoids (∼4 nm × ∼2 nm × ∼1.5 nm in size), with the N- and C-termini situated at opposite ends of the longest axis [34]. The 20 CCPs in the FH protein are connected to one another by short, sometimes flexible, linkers (of between three and eight amino acid residues) and arranged in an extended head to tail fashion, which makes the intact protein resemble a pearl necklace. As yet high-resolution 3D structures of the intact FH protein have not been generated, most likely due to its large size, glycosylation and inherent flexibility. Nevertheless, extensive investigations on recombinant constructs spanning individual/multiple CCPs of FH (either alone or in complex with ligands) have been carried out, and NMR and/or X-ray crystal structures are now available for all CCPs apart from 14 and 17 (see [13, 34, 36–41]). Despite a high degree of similarity in the 3D folds for these CCPs, different regions of FH display distinct functional activities (see Fig. 2), where it is now recognized that these originate from both diversity in the sequences of the individual CCPs (and thus differences in their functional surfaces) and from the different intermolecular interactions between neighboring CCPs that dictate their relative orientation [35]. Integration of these high-resolution data with lower-resolution structural information from small-angle X-ray scattering (SAXS) has provided insights into the overall organization/shape of the elongated FH molecule [34], e.g. such that CCPs 8–15 likely adopt a hinge like structure [36, 37, 42]; discussed in more detail below.

As highlighted in Fig. 1, FH possesses three complement regulatory activities: the protein (1) inhibits formation of the AP C3 convertase (C3bBb), (2) accelerates the dissociation of this complex, and (3) acts as a cofactor for the serine protease FI. FH does this by interacting directly with C3b, competing with FB for binding to C3b, thus inhibiting assembly of the C3 convertase and facilitating the decay of the already formed complexes (by displacing Bb from C3b). Furthermore, this interaction also makes C3b susceptible to cleavage by FI (i.e., to generate iC3b, an inactive form of C3b that does not bind FB). It is the 4 CCPs at the N-terminal end of the FH protein that mediate these inhibitory functions (see Fig. 2). This is now understood at a molecular level based on the crystal structure of C3b in complex with CCPs 1–4 of FH [41], where this and other structural studies have provided valuable insights into the functional biology of FH [34–40, 42, 43]. Importantly, these CCPs of FH bind to C3b in an extended configuration, with a remarkably long contact interface that covers the whole flank of the C3b protein [41]. This interface on C3b is generated during its transition from C3 (to which FH does not bind), and involves all four FH CCP modules and multiple regions of C3b, including the CUB module and thioester-containing domain (TED). The decay acceleration activity of FH, and inhibition of the formation of the AP C3 convertase, is mainly mediated by the first two N-terminal CCP domains of FH, which recognize a region of C3b that is involved in FB binding, e.g. dislocating Bb from C3b by electrostatic repulsion and via a steric hindrance mechanism. In addition, the interaction of FH with C3b provides a large contact surface for the binding of the serine protease FI bringing it into close proximity to the CUB domain of C3b (i.e., where the cleavages sites are located). Furthermore, given that the CUB domain is proteolytically cleaved when C3b is converted to iC3b, this study [41] also explains why CCPs 1–4 of FH do not bind iC3b [34]. The iC3b fragment generated retains the TED region and, therefore, when it is attached to a cell surface can undergo additional hydrolysis to smaller fragments (e.g., C3dg and C3d) by FI and membrane-bound cofactors (e.g., CR1). Importantly, C3d (essentially corresponding to just the thioester domain) is a ligand of CR2 on B cells, where this interaction is essential for lymphocyte activation and maturation [44].

The C-terminal two CCPs of FH (CCPs 19–20) have also been shown to be involved in FH’s interaction with the C3b (Fig. 2), where crystal structures of these CCPs in complex with C3d have been determined [39, 40]. These studies show that the binding site on FH mostly involves CCP19 along with the inter-domain linker between CCPs 19–20 (see [13]). Most importantly, from the superimposition of the X-ray structures of one of these structures [39] (where NMR and site directed mutagenesis allowed selection of the physiologically relevant interface) with that for C3b in complex with CCPs 1–4 [41] (described above) it is apparent that CCPs 19–20 recognizes a site on the TED domain that is close to, but distinct from, that occupied by CCPs 1–4. Thus, this strongly suggests that both ends of the FH protein (i.e., CCPs 1–4 and 19–20) can simultaneously bind the same C3b molecule [39].

Based on NMR studies on CCPs 10–11, CCPs 11–12 and CCPs 12–13, and SAXS modeling of CCPs 10–15 and CCPs 8–15 [36, 37, 42], it has been proposed that the middle region of FH (CCPs 8–15) adopts a hinge-like structure, with the CCPs 10–13 in a rigid V-like arrangement. Furthermore, combining these data with a SAXS-derived rod-like structure of CCPs 15–19 [39] and the crystal structures of CCPs 6–8 [45], and the CCPs 1–4 and 19–20 in complex with regions of C3, has allowed the generation of a model of FH where the protein bends back on itself [34]. Thus, this arrangement would provide a mechanism for how the N- and C-terminal ends of FH (i.e., CCPs 1–4 and CCPs 19–20) could bind simultaneously to distinct sites on the same C3b molecule. A key role in such structural organization is assumed to be played by CCP14 that is believed to alternate between poorly structured and compact conformations, depending on intra-molecular interactions (i.e., between neighboring CCPs) and ligand binding (i.e., to C3b associated with a non-activating surface) [37], thus allowing the two C3b binding regions of FH to swing around the central hinge region [34, 37]. Based on this hypothesis, a model has been proposed for the action of FH, where the protein is assumed to preferentially adopt a latent conformation with a low-affinity for C3b, which can switch to a higher-affinity activated structure upon recognition of C3b on *self* surfaces [34]. This transition has been proposed to be energetically driven by the binding of CCPs 1–4 to C3b and the interaction of CCPs 19–20 and CCPs 6–8 with glycans (such as glycosaminoglycans (GAGs) and sialic acid) on host surfaces, which would compensate for the loss of intra-molecular interactions stabilizing the latent conformation. As noted above, FHL-1 is identical to the full-length FH over the first 7 CCP domains and, therefore, contains the CCP 1–4 region associated with decay accelerating and cofactor activities (see [21]). However, given that FHL-1 lacks the sites for C3b binding and host surface recognition within the C-terminal region of FH (in CCPs 19–20), it is likely to be most effective as an inhibitor of complement activation within the fluid-phase [46].

## Interaction of factor H with glycans—discriminating self from non-self

The ability to discriminate between *self*, *non*-*self* and *altered*-*self* is perhaps one of the most striking and important properties of the immune system. In this regard, sialic acid-capped glycans and the GAG chains of proteoglycans are abundant on the cell surface and in the extracellular matrix of host tissues, where these structures function as *markers of self* [14, 31]. By interacting with these surface patterns, it is thought that FH can prevent excessive or inappropriate AP activation/amplification on host tissues; e.g., the interaction with sialic acid increases the affinity of FH for C3b on the mammalian plasma membrane, which enhances the cofactor and decay accelerating activities of FH [13, 47]. On the other hand, microbes (*non*-*self*) normally do not express high levels of these glycans and are, therefore, poor binders of FH, thus permitting amplification of the complement AP [48]; moreover the LP is activated via recognition of lipopolysaccharide on Gram-negative bacteria, lipoteichoic acid on Gram-positive bacteria and β-glucan on fungi [5].

As shown in Fig. 2, FH has two distinct glycan binding regions, where CCPs 6–8 have been shown to interact with GAGs alone, whereas CCPs 19–20 bind GAGs and sialylated sugars, i.e. containing Neu5Acα2-3Gal (see [13, 14, 31, 49, 50]). Recently, elegant structural work revealed that when FH is bound to C3b, a major portion of its CCP20 module is exposed and properly positioned (relative to the thioester attached TED region) to interact simultaneously with a sialylated ligand (or potentially GAGs) on the cell surface [13], i.e. supporting formation of a ternary FH-C3b-glycan complex. Furthermore, this study allowed the identification of key amino acid residues in FH CCP20 that bind the glycerol side chain and carboxyl group of Neu5Ac (i.e., the predominant form of sialic acid found in mammalian cells). Interestingly, CCP20 acts as a *hot spot* for HUS-associated mutations, which indeed modify the Neu5Ac-binding pocket and alter the overall affinity of FH for sialic acid [13], ultimately leading to the defective complement regulation observed in this pathology (see [25, 51]); see further details below.

The CCP 6–8 and CCP 19–20 regions of FH have different specificities for GAGs [49, 50]; the former interacts with heparan sulfate (HS) and dermatan sulfate (DS), while the latter binds to just HS. Furthermore, biochemical and bioimaging studies [49, 50] have revealed that these regions mediate the binding to different (and distinct) HS structures, thereby providing a possible mechanism of tissue specificity; see reviews [14, 31, 52, 53]. Here, it is important to note that GAGs are highly diverse molecules with a huge sequence repertoire (e.g., based on variable sulfation/domain structure [14, 31]); in the case of HS, this is referred to as the *heparanome*, with differences in HS structure from cell type to cell type and tissue to tissue. For example, in the human eye there is considerable diversity in both GAG structure and in the proteoglycan core proteins to which they attach within different layers of the retina [54, 55]. Because of this, and since many proteins interact with HS, it has been hypothesized that tissue-specific differences in HS structure may act as *post codes* (or area/ZIP codes) that help regulate protein binding to cell surfaces and to the matrix [31]. For example, FH and FP can both interact with HS but their specificities are distinct such that they do not compete for binding sites; this means that the local HS structure can potentially dictate the relative amounts of these negative and positive regulators of the AP that are associated with a surface, and thus whether complement activation is promoted or inhibited (see [52]).

As noted above the two GAG-binding sites in FH also have different specificities. In this regard, the CCP 19–20 region of FH is solely responsible for the anchoring of this protein to the glomerular basement membrane in the human kidney (partly through HS recognition) [49]; conversely, it is predominantly the CCPs 6–8 region that mediates the recruitment of FH in the human eye by supporting its binding to HS (and DS) on the RPE and Bruch’s membrane, that together form the outer blood-retinal barrier. Why these two regions of FH should be tuned to interacting with different GAG structures is not understood, but it does provide an explanation for why coding changes in CCPs 6–8 and CCPs 19–20 (that affect GAG binding), via mutations/polymorphisms in the *CFH* gene, are differentially associated as risk factors for AMD and aHUS, which are diseases of the eye and kidney, respectively [31, 49]. Furthermore, how these two glycan-recognition regions co-operate is not well established; utilizing two binding sites simultaneously (that has been termed *combinatorial recognition capacity*) would be expected to lead to a higher avidity of interaction (see [34, 49]), but what effect this would have on the conformation of the FH protein and its association with C3b has not been determined experimentally. An attractive speculation is that the interaction of CCPs 6–8 with GAGs could position CCP4 of FH proximal to the (*non*-*activator*) surface and thereby facilitate the binding of CCPs 1–4 with a C3b molecule attached to this surface via its thioester, while CCPs 9–18 adopt a conformation that allows simultaneous binding of CCP 19–20 with another glycan (i.e., either sialic acid or a GAG) close by on the surface [34]. Structural studies on the CCPs 6–8 region indicate that all three CCP modules are likely involved in HS binding, where there is a central GAG-binding surface in CCP7 and additional binding sites in CCP6 and in the linker between CCPs 7 and 8 [45, 53]; biophysical data indicate that these sites together may allow the binding of extended HS sequences, e.g. of 24 saccharides in length. Given that FHL-1 lacks CCP8 (and CCPs thereafter) and has a unique SFTL sequence attached to the C-terminus of CCP7 (Fig. 2) it is possible that its specificity may differ somewhat from the full-length protein; it is known that FHL-1 can bind to heparin (a highly sulfated form of HS), but as yet the characterization of its GAG-binding properties have not been reported [21].

The importance of the GAG-binding specificity to FH’s function in *self* recognition is evidenced by the Y402H polymorphism that changes a tyrosine to histidine at amino acid 402 within CCP7 altering the sulfation pattern recognized in HS [45, 50, 56]; the 402H variant of FH requires more highly sulfated HS for binding, whereas the 402Y form of the protein recognizes a broader range of HS structures. It is unusual that a single amino acid change has such a pronounced effect on HS binding but structural studies (involving NMR analysis and crystallography) indicate that the two FH variants likely use a different mode of interaction (see [45, 53]); evidence suggests that the histidine-402 can interact directly with a sulfate group on HS, whereas when a tyrosine is in this sequence position such an interaction is not possible. The 402H and 402Y variants of FH have differential ability to interact with HS within the human Bruch’s membrane, where the former binds less well presumably because the HS sequences it recognizes are rare compared to those bound by the 402Y form of the protein [53]. This is an important finding since the Y402H polymorphism has been found to be a major risk factor for AMD, a disease where dysregulation of the complement AP has been strongly implicated and where the pathology develops in the central part of the retina (the macula) at the interface of the Bruch’s membrane and RPE (see [30]); the restricted binding specificity of the disease variant of FH has been hypothesized to lead to its impaired association with the Bruch’s membrane, and because this extracellular matrix doesn’t have any other inhibitors of the AP, this would allow inappropriate complement activation driving disease progression [50, 53]. Moreover, recent work has shown that there is a substantial reduction in the amount of HS in human Bruch’s membrane with age (and a small, but significant, reduction in the level of HS sulfation) that likely explains the larger difference in the relative binding activities of the 402H and 402Y variants in old versus young tissues [57]; interestingly, similar changes in HS level and composition are not seen in a closely positioned region of the eye (i.e., the neurosensory retina). Thus, it can be envisaged that tissue-specific (and even more localized) changes in the amount and/or composition of glycans with age or disease would have a profound effect on the ability of FH to recognize *markers of self* and, thereby, affect its regulatory functions (see [14, 30, 31]). In addition to AMD, this is of relevance to a number of pathologies where there is dysregulation of the complement AP, including aHUS and DDD; see below for further detailed discussion.

## Other ligands of factor H

### Matrix proteins

The extracellular matrix contributes to all aspects of tissue/organ function, dictating the tensile strength and turgor of the tissue and directing cell phenotype and function both through the communication of mechanical cues and by acting as a reservoir and gate keeper for the myriad of extracellular signaling molecules. A remarkable example of matrix is that of articular cartilage, since it accounts for ~95% of the tissue volume; the cartilage matrix is composed of collagens and other fibril-forming proteins that provide tensile strength and elasticity as well as proteoglycans that hydrate the tissue (and thus give rise to its load-bearing properties) and also contribute to formation of the fibrillar network [58]. Importantly, the matrix has been found to play a major role in the regulation of both the adaptive and the innate immune system [59] and, as already described above, the *glycomatrix* (i.e., GAGs and sialic acid) serve to prevent inappropriate complement activation on host tissues [14]. However, in inflammatory diseases the dysregulated synthesis of matrix components and matrix breakdown not only leads to the activation of further inflammation via toll-like receptors (i.e. responding to these *damage*-*associated molecular patterns* [59]), but also can influence the complement system in a multitude of ways (see [60]). For instance, many components of the cartilage matrix have been implicated in the modulation of the complement system, e.g. in the context of rheumatoid arthritis, where several small leucine-rich proteoglycans (SLRPs) appear to play a role [60–63]; these matrix molecules, hidden in the cartilage matrix in healthy tissues, are released into the synovial fluid (e.g., as fragments) where they can interact with components of complement. In this regard, three SLRPs (fibromodulin, osteoadherin and chondroadherin) have been shown to be potent activators of the CP, via the selective recruitment of C1q [61, 62]. However, these proteins can also bind FH (and C4BP), where these interactions have been proposed to limit complement activation to the early steps of the CP [61–63]; interestingly, it is the CCP 6–8 region of FH that mediates the interaction with fibromodulin and chondroadherin, where in both cases the Y402H polymorphism reduces the strength of binding [61, 64]. The NC4 domain of type IX collagen, which is proteolytically released from cartilage during articular joint disease, can directly inhibit complement activation via its association with C3, C4 and C9, where for example the latter inhibits C9 polymerization and the formation of the membrane-attack complex [65]; it can also bind to FH and enhance FH’s cofactor activity for the FI-mediated breakdown of C3b. Furthermore, the NC4 fragment can also interact with fibromodulin and osteoadherin and inhibit their binding to C1q, illustrating the highly complex role of the matrix in the regulation of complement.

### Apoptotic and necrotic cells

To maintain tissue homeostasis, old and damaged cells undergo apoptosis and are replaced by new ones [66]. In the apoptotic process several changes occur in the morphology of the nuclear and plasma membrane that lead to recognition and phagocytosis of apoptotic cells by professional phagocytes; this includes several roles for complement, including opsonization (e.g., with C1q and C3b) and lysis of apoptotic cells [67, 68]. Rapid and efficient removal of apoptotic cells is clearly important to make space for living cells and to maintain tissue homeostasis; especially, since uncleared dying cells can undergo secondary necrosis, with the release of their intracellular contents, where this has been linked to autoimmune diseases (see [69]). During apoptosis the activation of caspases leads to the release of soluble chemo-attractants that can guide phagocytes towards apoptotic cells, where the discrimination of dying from live cells involves exposure of molecules on the cell surface and changes in charge and glycosylation patterns [70–73]. Importantly, FH can bind to molecules that become exposed on cells during apoptosis (and necrosis), including DNA, histones and annexin II (and possibly the altered glycocalyx), where it is believed that this serves to minimize the downstream pro-inflammatory effects of complement activation and the release of autoantigens that would trigger autoimmunity [62, 64, 68, 69]; FH binding may also compensate for the loss of some membrane-bound inhibitors of complement. In this regard, blocking of FH binding to apoptotic cells leads to increased opsonization with C3b and MAC-mediated lysis [69]. Three regions of the FH protein (CCPs 6–8, 8–15 and 19–20) have been implicated in the binding to apoptotic cells (whereas only CCPs 6–8 and 19–20 are involved in the association of FH with necrotic cells), where all of these regions mediate the binding to histones, the CCP 6–8 region mediates the interaction with annexin II and both the CCP 6–8 and CCP 19–20 regions bind DNA; the Y402H polymorphism affects the binding of FH to annexin II and DNA, impairing the former and enhancing the latter [64, 68]. Interestingly, FH did not bind to either GAGs or sialic acid on apoptotic cells revealing that FH uses a different mechanism of action compared to that described above for the recognition of healthy *host* cells.

As discussed in the section below, FH also interacts with pentraxins, e.g. C-reactive protein (CRP) and pentraxin 3 (PTX3), which are soluble pattern recognition molecules (PRMs) that recognize apoptotic and necrotic cells and recruit FH on to their surface [74–77].

### Pentraxins

Pentraxins, which can be subdivided into short and long forms, are characterized by a cyclic multimeric structure, and members of this family share a 200-amino acid pentraxin domain that is highly conserved in evolution [1, 78–80]. CRP and serum amyloid P component (SAP), which are pentameric and considered to be the prototypic short pentraxins, are both produced in the liver during inflammation (e.g. in response to IL-6); they play key roles in innate immune and tissue homeostasis, where they help remove cellular debris and matrix components [1, 80, 81]. PTX3 is a long pentraxin that was identified as an IL-1-inducible gene in endothelial cells and as a TNF-stimulated gene in fibroblasts [82]. PTX3 is produced locally (e.g. during inflammation) by various cell types, including vascular, stromal and innate immune cells [83]. While the short pentraxins CRP and SAP are composed primarily of a pentraxin domain, PTX3 has an additional ~160 residue domain at its N-terminal end [80, 84]. Both short and long pentraxins interact with components of the three complement pathways, including FH [74, 76] and C4BP [85, 86].

PTX3, which is an octamer composed of eight identical protomers [87], enhances the binding of FH to apoptotic cells, and thus the deposition of iC3b (see [76]). The binding of PTX3 to FH occurs through CCP7 and CCPs 19–20 (see Fig. 2), where these regions interact with the C-terminal and N-terminal domains of PTX3, respectively [76]. In this case, the Y402H polymorphism was reported to have no effect [76], however, FHL-1 is able to bind [88]. Interestingly, the glycosylation status of PTX3’s C-terminal domain has been shown to affect its interaction with FH, suggesting that tissue specific differences in this post-translational modification might play a role in tuning PTX3 activity [76, 89]. Furthermore, mutations in CCP 20, associated with increased risk for aHUS, have been reported to affect the interaction with PTX3, which has allowed the identification of a putative binding site on this terminal CCP of FH [88].

CRP cooperates with the complement system to remove apoptotic/necrotic cells [74, 90]. Indeed, it can activate both CP and LP by interacting with C1q and ficolins, respectively; this promotes complement-mediated opsonization and enhanced phagocytosis of target cells [91, 92]. In particular, CRP can bind to Fcγ receptors on leukocytes and thereby directly activate phagocytosis [93]. The interaction of FH with CRP [75, 94] has been shown to play important roles at sites of tissue damage and local inflammation, for example, in the atherosclerotic plaque [94]; the binding is believed to be mediated primarily by CCP7 with other sites in CCPs 8–11 (see [64, 95]) and CCPs 19–20 [75, 77]. Complement activation in the arterial intima can be initiated by components of both CP (i.e., C1q) and AP (i.e., C3b) that have been deposited on *activating surfaces*, such as immune complexes, CRP and oxidized LDL particles. When bound to CRP and cell surface glycans, FH down-regulates complement and protects the endothelium from MAC-mediated damage. Importantly, the FH Y402H polymorphism markedly reduces CRP binding capacity [64, 95], and could, therefore, lead to aberrant regulation of the complement cascade, e.g. in the context of AMD (see below). In other studies it has been proposed that it is the monomeric (pro-inflammatory) rather than pentameric (anti-inflammatory) form of CRP that interacts with FH and, in this way, limits complement activation, increases phagocytosis of apoptotic cells and reduces the inflammatory response by macrophages [77]. Interestingly, it has been observed that the 402H variant of FH has reduced binding to monomeric CRP as compared to the 402Y allele, and, therefore, in this context, may have an impaired anti-inflammatory activity [64, 96, 97].

### Cellular receptors

FH can bind several cell types and its functions go beyond the canonical role of a complement inhibitor [98]. For example, Avery et al. first identified complement receptor 3 (CR3, also known as CD11b/CD18 or integrin αMβ2) as a FH receptor on human neutrophils [99], where CR3 engagement by FH and FHL-1 modulates cell activation and function [100]; interestingly, recent data show that while FH can support neutrophil recruitment, it may also play an anti-inflammatory role, reducing tissue damage by influencing the formation of neutrophil extracellular traps [101]. Available data indicate that FH likely also binds CR4, possibly triggering a similar cellular response, e.g. on macrophages and dendritic cells that (unlike neutrophils) express significant amounts of CR4. Also new roles for the FH-CR3 complex have been proposed based on the observation that, when FH is bound to the yeast form of *C. albicans*, neutrophils strongly adhere and exhibit increased phagocytosis of this opportunistic fungal pathogen [99]. Therefore, in a sharp contrast to its role as a complement inhibitor, FH might promote the physical contact between neutrophils and certain pathogens, thereby increasing antimicrobial activity. Conversely, it has also been found that the FH-CR3 interaction can facilitate the entry of certain pathogens (i.e., *Streptococcus pneumoniae* and *Neisseria gonorrhoeae*) into host cells [102, 103].

In addition to CR3 and CR4, other membrane-bound molecules have been proposed as putative FH receptors, such as integrins (e.g. αIIbβ3) and L-selectin on human neutrophils, B lymphocytes, monocytes, and platelets [104–108]; as discussed below, thrombomodulin, a cell-surface thrombin-binding protein and regulator of the coagulation system, is also a ligand for FH on endothelial cells [109, 110]. Moreover, it has been observed that FH binds human B lymphocytes, thus inducing calcium-dependent FI release [105] and triggering blastogenesis [111]. Also, a chemotactic effect on monocytes has been reported for both intact [112] and thrombin-cleaved FH [113]. These additional interactions appear to mediate different functions to those involved in host recognition and complement regulation, ranging from the control of cell adhesion and migration to cytokine induction and a potential direct modulatory role on B cells. In this regard, FH might have a direct anti-inflammatory and tolerogenic activity towards infiltrated leukocytes, as suggested by the recent observation that monocyte-derived dendritic cells that have been treated with FH have lower expression of maturation markers and costimulatory molecules, decreased production of pro-inflammatory Th1-cytokines (i.e., IL-12, TNF, IFN-γ, IL-6, and IL-8), and preferential production of immunomodulatory mediators (i.e., IL-10 and TGF-β) [114].

### Miscellaneous ligands

FH has been identified as a binding partner for adrenomedullin (AM) (see [115, 116]), a regulatory peptide expressed in many tissues and cell types (including monocytes, macrophage, lymphocytes and granulocytes, and that is particularly abundant in the vascular endothelium), which has important physiological roles encompassing effects on vasodilatation, bronchodilatation, regulation of hormone secretion, control of apoptosis, angiogenesis and antimicrobial action [117, 118]. FH, studied under the name of AM-binding protein-1 [115, 116], has been found to enhance AM-mediated induction of cAMP in fibroblasts, augment the AM-mediated growth of a cancer cell line, and suppress the bactericidal capability of AM on *E. coli*. These activities are thought to be due to a stabilization effect of FH on AM, where FH inhibits the cleavage of AM by its candidate protease matrix metalloprotease 2 (see [116]) and co-administration of AM and FH reduces liver and kidney damage as well as systemic inflammation in a rat model of haemorrhagic shock [115]. Given the prominent roles of AM in blood pressure homeostasis, these data strongly encourage the targeting of AM with FH or FH-derived peptides/fragments as a pharmacological approach in the therapy of haemorrhagic shock and sepsis. In this regard, FH has been reported to interact with AM through a number of regions, where a high affinity site may be present in CCPs 15–20; see the excellent review by Sim et al. [116] for further discussion.

Aberrant accumulation of prion protein (PrP) is recognized as an important process in a number of neurodegenerative transmissible spongiform encephalopathies such as Creutzfeldt-Jakob disease in humans and bovine spongiform encephalopathy in cattle (see [119]). The complement system might play a role in the pathogenesis of these conditions, given that C1q, C3b and MAC are present in the PrP plaques of human brains with prion disease [120]; moreover, oligomers of human PrP can activate both the AP and CP. However, complement activation is restricted to its early stages (i.e., MAC formation initiated by PrP is at a low level in comparison to the deposition of C3b and C4b), where this has been ascribed to the ability of PrP to establish additional interactions with FH and C4BP [119]. Thus, complement might represent a target for neurodegenerative diseases associated with prions.

Additional ligands of FH include markers of oxidative stress, such as malondialdehyde (MDA) and malondialdehyde acetaldehyde (MAA) [121–123], and serum apolipoprotein E (ApoE) [124]. For example, FH is a major MDA-binding protein that interacts via CCPs 7 and 20 (see Fig. 2), where the Y402H polymorphism leads to substantially impaired binding [123]. Through its interaction with MDA, FH has been shown to bind to apoptotic and necrotic cells, inactivate complement (via its cofactor activity) on MDA-labeled surfaces, inhibit the uptake of MDA-modified proteins by macrophages and neutralize the pro-inflammatory effects of MDA in leukocytes and stromal cells, e.g. inhibiting the production of IL-8 in RPE cells. Thus, FH’s recognition of products of lipid peroxidation might help prevent excessive complement activation at sites of oxidative tissue damage and inflammation, e.g. in the context of chronic inflammatory diseases [125]. FH can also interact with ApoE, via CCPs 5–7 (see Fig. 2), on high density lipoprotein (HDL) particles in plasma and can thereby regulate/prevent complement activation [124].

## Factor H and immune evasion

Microbes have evolved several strategies to escape the immune response allowing their spread in the host [48, 126]. Given that the complement system is at the forefront of the immunological battle against invading microorganisms, it is not surprising that a number of its components have become the targets of major evasion mechanisms. For example, some microbes express proteolytic enzymes that inactivate C3b on their own surface or contain glycophosphatidylinositol-anchored complement inhibitors [127]. An additional way for pathogens to escape complement attack is by recruiting (i.e., *hijacking*) complement inhibitors from the host, such as FH (and C4BP) [128–130]. Indeed, some pathogens mimic a host surface by capturing these soluble complement regulators or, alternatively, target and* inactivate* complement inhibitors with secreted proteins/proteases [48, 131]; once a given pathogen has recruited complement inhibitors on to its own surface, it is likely protected from the complement attack as if it were *self*. There are several examples of bacteria that can acquire FH via the presentation of specific binding proteins. Amongst these are the factor H binding protein (fHbp) of *Neisseria meningitidis* [132], the outer surface protein E [133] and CspA [134] of *Borrelia burgdorferi*, Sbi (the staphylococcal binder of IgG) from *Staphylococcus aureus* [135] and the pneumococcal surface protein C of *Streptococcus pneumoniae* [129, 136, 137]. Despite the fact that these organisms are evolutionary distinct from each other and the FH ligands they express are structurally different, they all share a related binding site on FH that is localized on a common face of CCP20 [126]; these interactions enhance the binding of FH CCPs 19–20 to C3b, thus leading to a stable ternary complex between FH, C3b and the microbial protein, with increased co-factor activity (with conversion of C3b to iC3b). Thus, this *super*-*evasion* site, resulting from convergent evolution, provides further compelling evidence for the role of FH as a key regulator of the immune system.

An additional evasion site has been identified in the CCP7 domain, which mediates the recruitment of FH onto the surface of *Streptococcus pyogenes*, via the M protein, the main virulence factor of group A streptococcus (see [53, 138]). Interestingly, the Y402H polymorphism leads to reduced binding of FH to this organism and its increased phagocytosis in blood. These observations suggest that the presence of the 402H allotype might be protective against infections from group A streptococcus, which can be fatal in childhood, and might partly explain the high frequency of Y402H in some populations, e.g. ~30% of European-descended individuals carry the 402H allele [53]. The CCP 6–7 region (in addition to CCP20) has been described as a major binding site for fHbp of *Neisseria meningitidis*, however, the Y402H polymorphism does not affect this interaction [139]. Interestingly, the high affinity of fHbp for FH suggests that *N. meningitidis* could rapidly sequester FH from plasma, depleting its levels in circulation and allowing C3 turnover, a process that might contribute to the dramatic haemorrhagic rash seen in meningococcal sepsis. Other bacteria, for instance *Pseudomonas aeruginosa,* express sialic acid on their surface [140], where this is used to mimic a *self*-*glycan pattern* and recruit FH from the host’s blood, thereby escaping the complement-mediated immune response [141]. It has recently been suggested that the binding of FH to ApoE on HDL (via its CCP 5–7 region) provides an explanation for why it is beneficial for bacteria to bind to this part of FH [124]; i.e. by binding FH in this way bacteria are able to mimic plasma HDL increasing their survival in blood.

Similar *evasion strategies* have been developed by fungi. For example, the opportunistic fungus *Aspergillus fumigatus* has acquired the ability to recruit both FH and C4BP onto its cell wall [128], however, in this case the microbial ligands are unknown [142]. As noted above, some pathogens can escape the immune response by synthesizing and secreting soluble proteases that can target selected complement components. This is the case for *Candida albicans* whose aspartic protease 2 inactivates FH and the macrophage FH-receptors CR3 and CR4 [131]. In this context, it appears that the selective degradation of FH by the microbe-encoded protease helps circumvent the pro-phagocytic activity of FH (see above).

Although not directly related to the recognition of microorganisms, a noticeable example of FH-mediated immune evasion is represented by mosquitoes, which are important vectors in the transmission of many human diseases (e.g., malaria, dengue, yellow fever etc.) [143]. In this regard, human FH from a blood meal has been found to bind the luminal surface of the mosquito’s midgut and, via its cofactor activity, prevent complement activation on these surfaces, allowing the mosquito to tolerate the ingestion of large quantities of human blood. Interestingly, the merozoites from *Plasmodium falciparum* (i.e., the invasive form of the malarial parasite released on erythrocyte rupture) have been shown to evade the complement system by capturing FH and FHL-1 from serum (via the Pf92 protein) and down-regulating the AP [130].

The discovery of complement-evasion strategies employed by microorganisms, as described above, has led to the development of *complement vaccines* [48, 144]. Potential targets of these are the ligands of complement inhibitors made by bacteria; such that, if binding is prevented then complement activation would be expected to be promoted on the bacterial surface leading to its phagocytosis or lysis [48]. For example, recent progress has been made towards the development of a vaccine for *N. meningitidis* by targeting the factor H-binding protein fHbp [145]. Alternatively, engineered forms of FH might be used to elicit rapid and robust complement responses against specific pathogens. This is the approach that is forming the basis of a novel strategy for the treatment of the sexually transmitted infection gonorrhea, where the infectious agent *Neisseria gonorrhoeae* has developed resistance to almost every conventional antibiotic [146–148]. This bacteria escapes complement activation by interacting via its factor H binding protein with FH at sites within CCPs 6–7 and CCPs 18–20 [146]. Based on this, a chimeric protein comprising CCPs 18–20 of human FH fused to IgG Fc was designed, where key amino acids involved in FH binding to host cells were mutated while retaining its binding to gonococci [147]; this construct activated the CP and promoted a strong complement-dependent bactericidal activity. Recent work has incorporated a mutation into the CCP 19–20 region of the fusion protein that abolishes its binding to erythrocytes while retaining the interaction with *N. gonorrhoeae*, which promotes complement-mediated killing of the bacteria but without causing lysis of red blood cells [148]; in vivo testing in a mouse model of gonococcal infection has provided evidence that this has potential as a therapeutic against multidrug-resistant *Neisseria gonorrhoeae*.

## Factor H-associated diseases

FH plays a prominent role in the pathogenesis of several diseases in which genetic variations in the *CFH* gene are associated with modifying risk and/or where complement dysregulation is central to their etiology; these include typical and atypical HUS [27, 149, 150], AMD [30] and DDD [32, 33]. Furthermore, FH has long been regarded as a target for immune evasion by cancer cells [151], in a similar way to pathogens that hijack it to protect themselves from a complement-mediated response [126]. However, this paradigm has been recently challenged by the observation that reduced or impaired recruitment of FH to the tumor site can cause local inflammation, which in turn sustains rather than prevents cancerogenesis [152]; see further discussion below.

### Hemolytic uremic syndrome

HUS is the most common cause of acute renal failure amongst children and its clinical symptoms are often a combination of hemolytic anemia, thrombocytopenia and acute renal failure [153]. This disease is classified as either typical or atypical and referred to as HUS and aHUS, respectively. HUS is characterized by bloody diarrhea caused by verotoxin/shiga toxin producing enterohaemorrhagic *E. coli* (EHEC) [27]. The pathogenesis of this form of HUS is still poorly understood, however, it has been reported that shiga toxin causes damage to endothelial and epithelial cells by inhibiting protein synthesis and inducing apoptosis [154]. Importantly, this toxin activates complement, mostly via AP in the fluid-phase, and delays the cofactor activity of FH on the cell surface, thus exacerbating complement-dependent tissue damage [155]. Shiga toxin interacts with FH at sites within CCPs 6–8 (and CCPs 18–20) where the 402Y variant of FH had somewhat higher binding activity compared to the 402H form, such that individuals carrying the former allele might be more vulnerable to HUS. Furthermore, a direct interaction between shiga toxin and FHL-1 has been described, indicating that this truncated variant of FH might also have a role in the pathogenesis of EHEC-associated HUS [26].

aHUS is not caused by an infection, but instead is directly associated with FH mutations that impair recognition of *self* surfaces [13, 156]. As described above, the C-terminal CCP20 domain of FH is known to mediate interactions with *glycan markers* on certain host cells (e.g., endothelial cells) and in the matrix, and in this way provides protection from autologous complement activation [49]. Most aHUS-associated FH mutations map to this region of the protein (see [13]), and have been described to impair cell binding and cell surface protection from complement attack, thus contributing to endothelial injury and microvascular thrombus formation, with major clinical manifestations at the level of the central nervous system and kidney [40, 156]; indeed, recent work has revealed that certain aHUS mutations impair the recognition of sialic acid by FH on endothelial cells, erythrocytes and platelets, indicating that sialic acid (rather than HS) is critical for FH-mediated complement regulation on these cell types [157]. Furthermore, many patients with aHUS have FH autoantibodies that are directed against the C-terminal domain (i.e. CCP20), thus inhibiting the binding of FH to host surfaces [158–160].

In addition to FH, mutations in other genes have been reported to be associated with aHUS, including C3, FB, FI, MCP and thrombomodulin [156, 161, 162]. Thrombomodulin is a cell surface FH-binding protein that enhances FH cofactor activity probably through stabilization of the FH-C3b complex on endothelial cells [110], where aHUS-associated mutations in thrombomodulin interfere with the FH interaction [109]. Thus, thrombomodulin, in addition to its classical role as an inhibitor of the coagulation system, also provides an alternative mechanism by which FH can protect endothelial surfaces.

### Dense deposit disease (DDD)

Dense deposit disease, formerly known as membranoproliferative glomerulonephritis type II, is a renal disorder characterized by unchecked AP activation in the plasma and high levels of C3 deposition on the glomeruli, with severe impairment of the kidney’s draining functions [32, 33]; as its name suggests electron-dense intramembranous deposits form within the glomerular basement membrane, a specialized extracellular matrix of the kidney. Several genes have been associated with this pathology, and most of them code for components of the complement system, including FH [28, 33]. DDD-associated mutations in the *CFH* gene mainly affect FH protein folding and secretion, and its cofactor activity [163, 164]; for example, a mutation causing the replacement of a cysteine with a tyrosine at position 431 (in CCP7) induces protein aggregation [163], whereas mutations in CCP4 lead to impaired cofactor activity [41]. DDD (and aHUS) can also be caused by total deficiencies in FH production, where the defective control of AP activation leads to C3b deposition in kidney glomeruli and a secondary deficiency in C3 (due to its uncontrolled turnover) (see [165]). Furthermore, autoantibodies against FH can also be associated with DDD where these block formation of the C3b/FH complex, providing another mechanism for uncontrolled complement activation [166, 167].

### Age-related macular degeneration (AMD)

Age-related macular degeneration is the most common form of vision loss in the developed world. Indeed, it is predicted that by the year 2020, 196 million people worldwide will suffer from some form of the disease [168]. The condition manifests itself in the central part of the retina, called the macula, resulting ultimately in the complete loss of central vision. Late-stage (advanced) AMD is divided into two forms: *wet* and *dry*. In the former, there is growth of new (leaky) blood vessels from the choroid across the RPE and Bruch’s membrane into the retina and in the latter there is loss of cells and tissue structure in the choroid, RPE and neurosensory retina [30, 169]. Late-stage AMD is usually preceded by the formation of deposits called *drusen*, which contain complement activation products, that form on/in the Bruch’s membrane; as noted above this is an extracellular matrix that along with the RPE forms the outer blood-retinal barrier, allowing the transport of nutrients and waste products into and out of the retina while preventing the migration of leukocytes to this immune privileged site.

AMD is a multifactorial disease where there is a large genetic component, along with age, diet, and smoking all being additional risk factors [170]. A large number of genetic variants that alter the risk for developing advanced AMD have been identified through numerous genome-wide association studies (see [171, 172]); importantly, the majority of the strongest associations reside in genes of the complement system [172, 173]. Of these, the Y402H polymorphism in *CFH* is considered to represent the greatest (single) genetic risk factor of developing AMD [29, 170]. As described in detail already, this polymorphism affects the coding sequence of CCP7 in the full-length FH protein and FHL-1, since they are both produced by alternative splicing of the *CFH* gene; the altered specificity for GAGs (e.g., in the human Bruch’s membrane) as well as changes to other binding functions (e.g., CRP and apoptotic/necrotic cells) are of likely relevance to the pathology of AMD, where local (chronic) inflammation, driven by complement dysregulation, is thought to be of central importance [30, 31, 174].

In the case of FHL-1, which has been shown recently to be the predominant *CFH* gene product associated with Bruch’s membrane [21], CCP7 represents the only GAG-binding domain for the protein. Therefore, it is likely that FHL-1 is more susceptible to alterations in GAG recognition conferred by the Y402H polymorphism (although no data are yet published on this). Indeed, Bruch’s membrane from human donor eyes [175] failed to retain as much of the AMD-associated 402H variant of FHL-1 compared to the 402Y form when the proteins were individually diffused across this ECM [21]. The finding that the Y402H polymorphism has no effect on systemic levels or activity of FH/FHL-1 [176] is consistent with AMD being a disease of local complement dysregulation, e.g. at the interface of the RPE and Bruch’s membrane, which is where the pathology initially develops.

### FH and cancer

There is increasing evidence that complement plays a role in cancerogenesis and in the tumor microenvironment [177]. Membrane-associated inhibitors of the complement system, such as DAF, MCP, CR1 and CD59, are highly expressed on certain tumors, which likely help cancer cells to escape complement activation. Similar strategies are adopted by tumor cells that express and release high amounts of FH, contributing to reduced complement activity in the tumor microenvironment [178]; in these cases, FH has indeed been proposed as a new cancer marker [179]. Interestingly, cancer cells also display an increased amount of sialic acid, and sometimes GAGs, on their surfaces, where this *altered self* (or *super self*) is believed to be able to recruit FH and provide protection against complement-mediated lysis (see [14]). Interestingly, sera from patients with early stage, non-metastatic, non-small cell lung cancer were found to contain autoantibodies against FH, where these recognize a conformationally distinct (reduced) form of the protein that is believed to be displayed only on the surface of tumor cells [180]; the autoantibodies interact with CCP 19 and inhibit the binding of FH to lung carcinoma cells, thereby promoting the deposition of C3b and increasing complement mediated tumor cell lysis. Furthermore, a recombinant anti-FH autoantibody has been demonstrated to activate complement on tumor cells, leading to the release of anaphylatoxins that induce complement-dependent cytotoxicity and inhibit tumor growth in vivo [181]. These recent findings provide an exciting opportunity to develop a novel immunotherapeutic strategy to treat cancer by targeting FH.

**Fig. 3:**
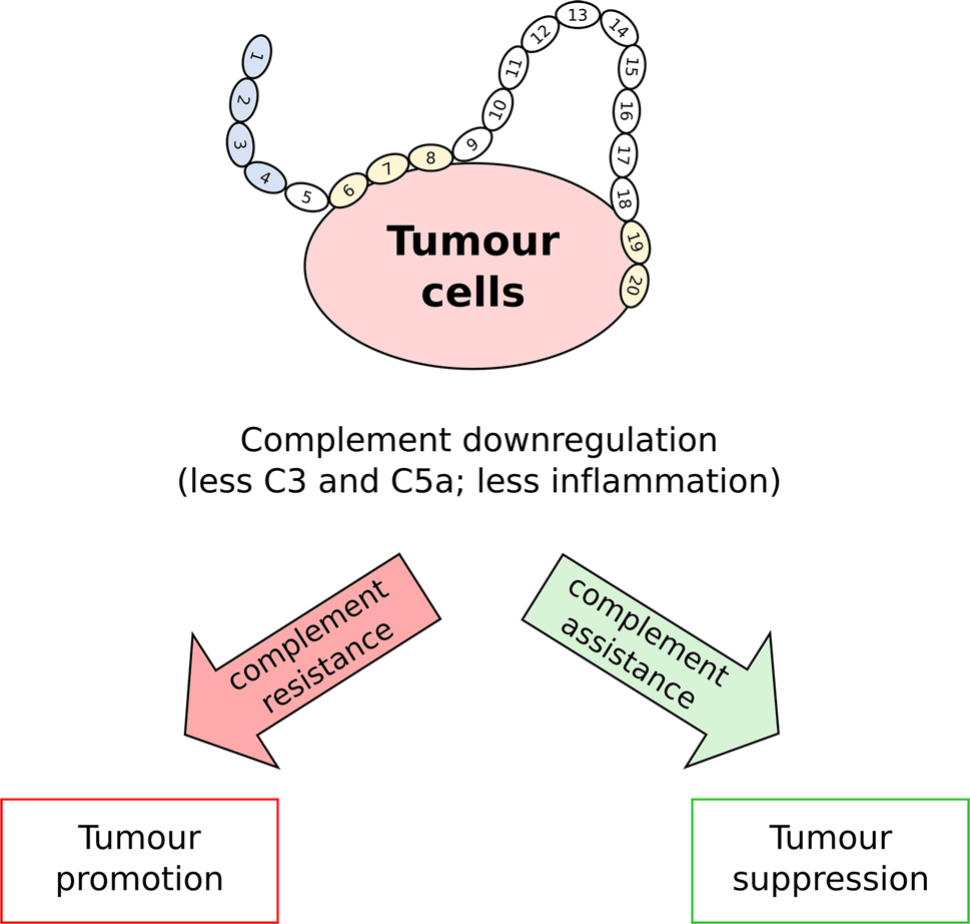
A dual role for FH in cancer. FH is expressed or recruited by cancer cells and this leads to reduced deposition of C3 and less production of the anaphylatoxin C5a. Inhibition of complement attack (*complement resistance*) can favor tumor survival and promote tumor growth (*tumor promotion*). However, in certain types of cancer that are inherently promoted and sustained by inflammation, the anti-inflammatory microenvironment generated by FH (*complement assistance*) might be unfavorable to tumor growth and progression (*tumor suppression*)

According to the traditional paradigm, complement is *good*, in that it recognizes, when not deceived, the cancer cell, and either directly (via MAC-mediated lysis) or indirectly (via complement-dependent cell toxicity) kills or disposes of it. New ideas, however, are emerging that challenge this and point to the complement system as a factor in the tumor’s ability to promote inflammation [152]. For example, in a mouse model of chemically induced carcinogenesis, the genetic deficiency of PTX3 (a ligand of FH; see above) causes an increase in both growth and size of the tumor lesions; this has been ascribed to defective recruitment of FH onto cancer cells in the *PTX3* ^−*/*−^ mice. In fact, higher levels of C3 and C5a have been described in tumors from the PTX3-deficient animals, suggesting that complement regulation in the absence of this long pentraxin is subverted. These data indicate that the complement system might promote cancer-related inflammation, and in this way support rather than oppose cancerogenesis and tumor growth [152] (see Fig. 3). An active role of complement in tumor growth and metastasis has been proposed in other studies. For example, upon complement activation, the anaphylatoxins C3a and, in particular, C5a initiate and modulate the inflammatory response by mediating chemotaxis, inflammation and generation of reactive oxygen species (ROS) [182]. The resulting microenvironment appears to favor cancerogenesis as well as tumor immune escape and progression [183]. Thus, FH appears to have opposing roles in cancer and whether it promotes an unfavorable environment for cancer through its anti-inflammatory properties (*complement assistance*) and/or is hijacked by cancer cells to provide immune evasion (*complement resistance*) likely depends on the type/stage of cancer involved; clearly there is further work needed to more fully understand FH’s exact role in cancer immunology.

## Concluding remarks

In this review we discussed the structure/function relationships of complement FH, with a focus on the emerging roles of this protein in the pathophysiology of infectious diseases, inflammatory conditions and cancer. FH is the major soluble inhibitor of the AP C3 convertase, an enzyme complex that is at the very center of the complement cascade. Therefore, alterations of FH protein structure, due to mutations and polymorphisms, likely lead to aberrant functional outcomes. This is the case for FH mutations associated with aHUS, AMD and DDD all diseases of local complement dysfunction where a loss of appropriate self-recognition, e.g. within the extracellular matrix, appears to be a common factor. Also, FH is a preferential target for complement evasion by a number of pathogens. This has paved the way towards complement vaccines, which are being designed and developed based on FH microbial ligands, and novel FH-based therapeutics. Finally, a dual role is emerging for FH in cancer, where it can either be hijacked by cancer cells to avoid the complement attack, or can be used to dampen cancer-related inflammation.
